# Connecting Patients with Clinical Trials Using Patient Navigation: A Scoping Review

**DOI:** 10.3390/curroncol33060341

**Published:** 2026-06-08

**Authors:** Olla Hilal, Ria Patel, Pratham Gupta, Nicole Askin, Victoria Ivankovic, Carla Epp, Renee Nassar, Milica Paunic, Mahmoud Hossami, Rhonda Abdel-Nabi, Michael Touma, Govana Sadik, Anaam Jaet, Christina Trieu, Ibrahim Mohamed, Gregory Anagnostopoulos, Leonard Yoo, Mohammad El Hindawi, Caroline Hamm, Megan Delisle

**Affiliations:** 1 Department of Epidemiology and Biostatistics, Western University, London, ON N6A 3K7, Canada; ohilal@uwo.ca; 2 Schulich School of Medicine & Dentistry, Western University, London, ON N6A 3K7, Canada; rpate455@uwo.ca (R.P.); pgupta2029@meds.uwo.ca (P.G.);; 3 WRHA Virtual Library, University of Manitoba, Winnipeg, MB R3E 3P5, Canada; 4 Department of Surgery, University of Ottawa, Ottawa, ON K1N 6N5, Canada; 5 Windsor Regional Hospital, 1995 Lens Ave, Windsor, ON N8W 1L9, Canada; 6 Temerty Faculty of Medicine, University of Toronto, Toronto, ON M5S 1A1, Canada; milica.paunic@mail.utoronto.ca; 7 University of Windsor, Windsor, ON N9B 3P4, Canada; 8 Department of Surgery, University of Manitoba, Winnipeg, MB R3A 1R9, Canada; 9 Paul Albrechtsen CancerCare Manitoba Research Institute, 675 McDermot Ave, Winnipeg, MB R3E 0V9, Canada

**Keywords:** patient navigation, clinical trials, cancer, trial enrollment, health equity, access to care, scoping review

## Abstract

Patient navigation is a promising intervention to address barriers and improve access to cancer clinical trials. Although navigation has been widely studied across the cancer continuum, its role in facilitating clinical trial participation has not been systematically evaluated. This scoping review aims to identify, characterize, and synthesize evidence on patient navigation interventions designed to increase access to cancer clinical trials. Nine databases were searched for English peer-reviewed articles from inception through 5 March 2025. Two independent researchers screened titles, abstracts, and full texts and extracted data using standardized forms. The results were interpreted using descriptive statistics and collated using predetermined conceptual frameworks. Of 10,238 citations identified, 23 studies met inclusion criteria. All were conducted in North America, and five (21.7%) were randomized controlled trials. Thirteen studies (56.5%) evaluated enrollment outcomes, with mixed results. One randomized trial and two observational studies found no significant effect, while three observational studies and seven single-arm reports suggested improved enrollment. Navigation interventions most commonly included education/information provision (100%), care coordination (60.9%), and empowerment (47.8%). Navigators were primarily lay navigators (73.9%), often selected for community or lived experience; training varied widely, and only two programs reported certification. Fifteen studies (65.2%) targeted equity-deserving groups, most frequently racial and ethnic minorities, with reports of increased representation in trial enrollment. Patient navigation shows promise in improving access to cancer clinical trials, particularly for equity-deserving populations, but current evidence is limited, heterogeneous, and largely observational. Standardized definitions, rigorous trial designs, and reporting of navigator training and outcomes are needed to clarify effectiveness.

## Simple Summary

Clinical trials are important for improving cancer care, but many patients face barriers to learning about, accessing, and enrolling in trials. Patient navigation is a supportive approach where trained navigators help patients overcome barriers by providing information, coordinating care, addressing practical concerns, and connecting patients with appropriate services. This scoping review examined existing studies on patient navigation programs designed to improve access to cancer clinical trials. We found that navigation may help improve trial awareness, referrals, and enrollment, particularly for patients from equity-deserving groups, but the evidence remains limited and inconsistent. Most studies were observational, used different types of navigators and interventions, and did not consistently report training, outcomes, or long-term implementation. Future studies should use stronger research designs, standardized reporting, and patient-centered models that help connect patients to suitable trials across institutions. These findings may inform future research, program development, and policy efforts to improve equitable access to cancer clinical trials.

## 1. Introduction

Clinical trials are essential to advancing cancer care, yet persistent barriers to participation result in only 3–5% of adult cancer patients enrolling in trials [1,2,3]. Low enrollment limits the generalizability of trial findings, slows the development of new therapies, and may worsen inequities in access to innovative cancer care.

Barriers to clinical trial participation occur at multiple levels. At the patient level, individuals may be unaware of available trials, have limited understanding of trial processes, experience mistrust, or face language, cultural, financial, transportation, or caregiving barriers. At the provider level, clinicians may have limited time, incomplete knowledge of available trials, or uncertainty about eligibility criteria. At the health system and institutional level, trial access may be constrained by referral pathways, availability of research staff, geographic distance from trial sites, and differences in institutional trial portfolios. Trial-level barriers may include restrictive eligibility criteria, complex consent processes, time-sensitive enrollment windows, additional testing requirements, and costs or travel demands associated with participation. These barriers may be particularly pronounced for equity-deserving populations, including racial and ethnic minority groups, lower socioeconomic status populations, and geographically isolated communities. Strategies that help patients identify, understand, and access appropriate trials are therefore needed to improve both overall enrollment and equity in trial participation.

Patient navigation is one promising strategy to address these barriers. Patient navigation has been defined as an individualized intervention that aims to address barriers and facilitate timely access to health care services, diagnosis, treatment, and care [4]. Navigation commonly includes patient-centered strategies such as education, care coordination, advocacy, logistical support, financial assistance, and emotional support. Existing evidence suggests that patient navigation can improve access across the cancer continuum, including screening, diagnosis, and treatment. However, less is known about its role in improving access to cancer clinical trials specifically [5,6,7].

Navigation for cancer clinical trials presents challenges that differ from navigation for routine cancer screening or treatment. Clinical trial navigation may require identifying appropriate trials, interpreting complex eligibility criteria, supporting time-sensitive treatment decisions, and helping patients understand consent processes that may involve randomization, uncertainty, or additional testing. These challenges are increasingly important in the era of precision oncology, where trial matching may depend on molecular tumour characteristics and where suitable trials may not be available at a patient’s treating institution. As a result, navigation approaches that are effective in other areas of cancer care may need to be adapted for clinical trial settings.

Although patient navigation is increasingly being used to support clinical trial access, the evidence specific to this context has not been comprehensively synthesized. Important knowledge gaps remain regarding the design of navigation interventions, the qualifications and training of navigators, the outcomes used to evaluate these programs, and the extent to which navigation improves access for equity-deserving populations. Therefore, this scoping review aims to identify, characterize, and synthesize the available evidence on patient navigation interventions designed to increase access to cancer clinical trials. Specifically, this review characterizes existing interventions, summarizes reported outcomes, examines navigator training and qualifications, and identifies gaps to inform future research, program development, and policy.

## 2. Methods

### 2.1. Protocol and Registration

Our scoping review protocol was registered on Open Science Framework (https://doi.org/10.17605/OSF.IO/BTMYK) and published in a peer-reviewed journal [8]. A preliminary search of MEDLINE, the Cochrane Database of Systematic Reviews, and JBI Evidence Synthesis was conducted, and no existing or ongoing systematic reviews or scoping reviews on the topic were identified [9,10]. The search strategy can be found in Supplementary Table S1. Our review was guided by the JBI methodology for scoping reviews and reported according to the PRISMA extension for scoping reviews (Supplementary Table S2) [10,11].

### 2.2. Review Questions and Eligibility Criteria

The primary research question guiding this scoping review was: What is the existing evidence on patient navigation aimed at increasing access to clinical trials, and how are these characterized in terms of their design, implementation, and outcomes? In addition, two secondary questions were explored: What qualifications (e.g., certification, clinical background) and training do navigators have? and How does patient navigation address and impact access to clinical trials for equity-deserving populations (i.e., racial/ethnic minorities, lower socioeconomic status populations, or geographically isolated communities)?

The PCC (Population/Concept/Context) framework, as recommended by JBI, was used to develop our study eligibility criteria. The population included individuals who engage with navigation services in cancer care, such as patients, caregivers, and healthcare professionals. Although the primary focus of this review was patient access to cancer clinical trials, caregivers and healthcare professionals were included within the Population component because they may participate in, deliver, refer to, or evaluate patient navigation interventions. Studies involving caregivers or healthcare professionals were eligible only when the intervention or outcome was directly related to patient navigation for cancer clinical trial access. The concept focused on patient navigation in cancer clinical trials. The context included studies examining the use of navigation to increase participation in cancer clinical trials. Eligible study designs included experimental, quasi-experimental, observational, and qualitative studies. Systematic reviews and opinion papers were excluded to focus on the detailed findings of individual interventions.

### 2.3. Definitions

As previously mentioned, we adopted the definition of patient navigation developed by Chan et al. (2023), which defines patient navigation as “an individualized intervention that aims to address barriers and facilitate timely access to healthcare services, diagnosis, treatments and care” [4].

For the purposes of this review, patient navigation was distinguished from related concepts, including care coordination and case management. Care coordination was considered one component of patient navigation, rather than an interchangeable term. In this context, care coordination refers primarily to the organization of services and communication between patients, healthcare professionals, and care settings [4]. Patient navigation was conceptualized more broadly as a patient-centred, barrier-focused intervention in which a navigator supports individuals as they move through the healthcare system, including through education, emotional support, and strategies that promote self-management [12,13]. Case management was considered a related but distinct approach that generally emphasizes assessment, planning, coordination, and monitoring of health and social service needs. Therefore, for this review, studies were considered eligible when the intervention included individualized support to address barriers to cancer clinical trial access, rather than administrative coordination alone.

We categorized navigators as either lay navigators or nurse navigators based on the Oncology Navigation Standards of Professional Practice [14]. Lay navigators are “professionals who provide individualized assistance to patients and families affected by cancer to improve access to healthcare services. A [lay] navigator may be employed by a clinic or a community-based organization and work throughout the community, crossing the clinic threshold to continue to provide a consistent person of contact and support within the healthcare system. A [lay] navigator does not have or use clinical training” [14]. Nurse navigators are “professional registered nurses with specific clinical knowledge who offer individual assistance to patients, families, and caregivers to help overcome healthcare system barriers. Using the nursing process, a nurse navigator provides education and resources to facilitate informed decision-making and timely access to quality health and psychosocial care” [14].

### 2.4. Search Strategy and Information Sources

Eligibility was restricted to English-language publications. This decision reflected the review team’s available language expertise and the need for consistent interpretation of terminology related to patient navigation, care coordination, and case management during screening and data extraction. Non-English publications were not included because translation and validation of translated terminology were beyond the scope and resources of this review.

Articles published since database inception were included until the search was performed on 5 March 2025 (see Supplementary Table S1). The search strategy identified both published and unpublished studies. We first conducted a preliminary search in MEDLINE and CINAHL to identify relevant articles and refine the search terminology. Keywords from relevant titles and abstracts, together with database-specific subject headings, were used to develop the full search strategy. The final strategy was applied to Cochrane CENTRAL (Ovid), MEDLINE (Ovid), EMBASE (Ovid), CINAHL via EBSCOhost, Epistemonikos, and PROSPERO. Additional searches were conducted in Turning Research into Practice, the World Health Organization International Clinical Trials Registry Platform, Google Scholar, and the Agency for Healthcare Research and Quality. The search was structured around two core concepts: navigation, including related terms such as navigator, care coordination, and case management, and clinical trials. Search terms and syntax were modified as needed for each database or information source. The strategy was reviewed using the PRESS Peer Review of Electronic Search Strategies 2015 guideline [15].

The reference lists of all included articles were screened for additional relevant articles. Sources of unpublished studies and grey literature were searched separately, including conference abstracts, trial registries, thesis repositories, institutional databases, preprint servers, government reports, and professional organization websites.

### 2.5. Selection of Sources of Evidence

DistillerSR software (version 3.5) (Evidence Partners Incorporated, Ottawa, ON, Canada) was used for the screening. Identified citations were collated and uploaded into DistillerSR, where duplicates were removed. Level 1 (title and abstract) and level 2 (full-text screening) were conducted by two independent reviewers.

A pilot test was conducted, where each reviewer performed a level 1 screen on 10% of randomly selected citations. These were then compared and disagreements were resolved through discussion to align the reviewers. Following the pilot test, the remaining level 1 and 2 screens were conducted by two independent reviewers. Disagreements that arose between reviewers at each level of screening were resolved through discussion or with the assistance of an additional third reviewer. Reasons for the exclusion were recorded during level 2 screening.

Previous work by Tsou et al. supports the use of artificial intelligence (AI) tools for reviews with more than 2500 citations [16]. DistillerSR artificial intelligence tools were used to support the screening process. Screening results were cross-checked using the Check for Screening Errors tool, which identifies excluded citations that may warrant reassessment based on patterns learned from prior reviewer decisions [17]. The tool uses a machine-learning model with 10-fold cross-validation, in which the reviewed citations are divided into ten subsets. During each iteration, the model is trained on nine subsets and tested on the remaining subset, with the process repeated so that each subset is used once for testing [18]. This approach supports internal validation of the model and helps identify records that may have been incorrectly excluded [18].

We also used the Screening Prioritization tool, which applies machine-learning algorithms trained on reviewer-screened citations to rank remaining titles and abstracts according to their predicted likelihood of meeting inclusion criteria. This allowed the review team to use a stop-screening approach during level 1 title and abstract screening, whereby screening was discontinued once the prespecified estimated recall threshold was reached. A ‘stop screening approach’ is one where the review team stops screening when the number of citations falls below a specified probability for inclusion [19]. This is referred to as the ‘estimated recall rate,’ which can be calculated using DistillerSR’s AI tools after at least 2% of the total number of citations have been screened and conflicts resolved [19]. In this scoping review, an estimated recall rate of 95% was used based on prior recommendations [19]. The estimated recall rate represents the proportion of potentially relevant citations predicted to have been identified through screening. Therefore, a 95% estimated recall threshold indicates that the model predicts that approximately 95% of relevant citations have been captured among the screened records. We selected this threshold based on prior evaluation of DistillerSR’s prioritization tool by Hamel et al., who found that use of a 95% recall target substantially reduced missed studies while improving screening efficiency [19]. This approach allowed the review team to discontinue title and abstract screening after reaching the prespecified recall threshold, while maintaining a conservative approach to study identification [19]. Accordingly, not all records remaining after deduplication were screened at the title and abstract level, and the number of records screened reflects those reviewed prior to reaching the predefined recall threshold.

Cohen’s κ coefficient was used to calculate inter-rater reliability for the level 1 screening. Cohen’s κ coefficient assesses the degree of agreement between independent reviewers without any discussion [20]. Cohen’s κ greater than 0.80 is indicative of satisfactory agreement between reviewers [21,22]. Cohen’s κ coefficient ranged between 0.87 and 0.97 for all reviewers for level 1 screening.

### 2.6. Data Charting

The data abstraction tool was primarily structured using TIDieR (Template for Intervention Description and Replication), as the main purpose of this review was to characterize the design and delivery of patient navigation interventions. TIDieR informed extraction of intervention-level details, including the intervention rationale, materials, procedures, provider type, mode of delivery, timing, dose or intensity, and tailoring of the navigation intervention [23].

RE-AIM (Reach, Effectiveness, Adoption, Implementation, Maintenance) and PRISM (Practical Robust Implementation and Sustainability Model) were used as supplementary frameworks to capture implementation- and context-related information when reported [24,25]. RE-AIM guided extraction of information related to the target population and reach of the intervention, reported effectiveness outcomes, adoption setting, implementation features, and maintenance or sustainability. PRISM guided extraction of contextual factors, including intervention setting, delivery structure, patient population, and organizational or system-level considerations. Chan et al.’s patient navigation domains were used to classify the specific navigation functions delivered, including care coordination, education/information provision, empowerment, comfort/emotional support, direct care provision, advocacy, language assistance, logistics assistance, and financial assistance [4].

Outcomes of patient navigation, including trial knowledge, referral, enrollment, completion, satisfaction, and equity-related outcomes, were extracted when reported. Because included studies varied substantially in reporting detail, not all framework domains could be extracted for every study.

### 2.7. Synthesis of Results

We used qualitative content analysis to synthesize the extracted data and address the review questions. This approach allowed us to organize intervention characteristics, implementation features, navigator attributes, and reported outcomes into descriptive categories [22,26]. Coding was primarily deductive, with an initial coding structure informed by the review questions, established implementation frameworks, and patient navigation domains [4,22,27]. Additional categories were refined during extraction when study details did not fit the initial coding structure. The codebook specified coding categories for study characteristics, navigation intervention components, navigator type and training, timing and mode of delivery, implementation context, outcomes, and equity-related considerations [4]. When appropriate, categorical or short-text extraction fields were used to support frequency counts across studies [22,23,24,25,28,29].

Two reviewers independently extracted and coded data from included studies using the standardized abstraction form. When extracted data did not clearly fit an existing category, reviewers discussed whether the information should be coded under an existing domain or whether an additional descriptive category was required. Coding discrepancies were resolved through discussion, and unresolved conflicts were reviewed by a third member of the research team. Reliability of the synthesis process was supported through duplicate extraction and coding, use of a predefined codebook, pilot testing of the abstraction form, and consensus-based conflict resolution. Formal inter-rater reliability statistics were calculated for level 1 screening, as described above, but were not calculated for the qualitative synthesis because coding was used to descriptively map study characteristics rather than to generate independently rated outcome judgments. Data are presented in tables, figures and a narrative description to summarize the available evidence and results [22].

### 2.8. Critical Appraisal

Formal risk-of-bias assessment was not performed. This decision was consistent with the methodological approach for scoping reviews, which aim to map the extent, range, and nature of available evidence rather than to determine pooled intervention effectiveness. Given the expected heterogeneity in study designs, populations, navigation interventions, and outcomes, the review focused on describing study characteristics, intervention components, outcomes, and evidence gaps rather than excluding or weighting studies based on methodological quality.

## 3. Results

### 3.1. Sources of Evidence

**Figure 1:**
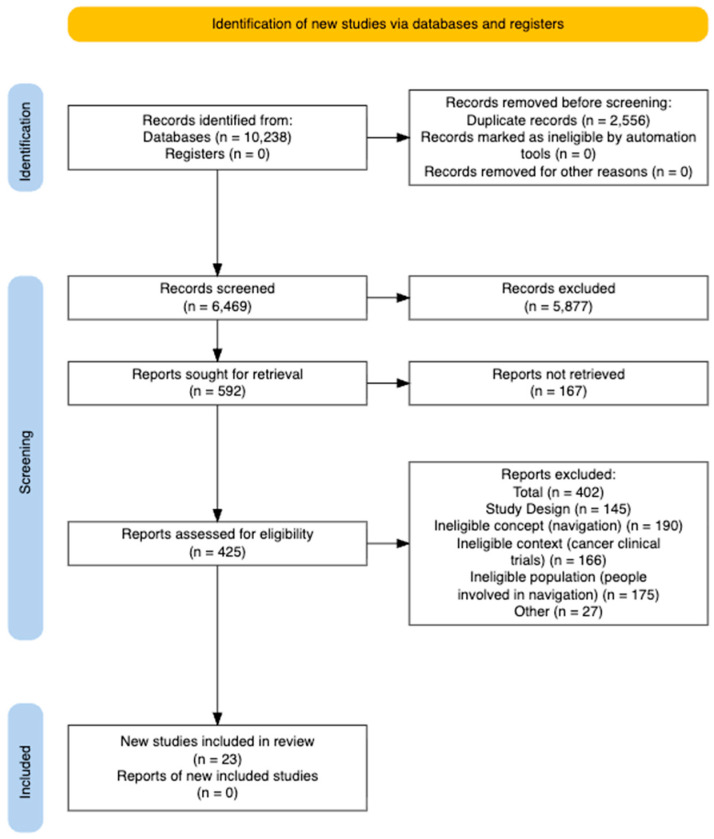
PRISMA Flow Diagram [30]. Note: Based on recommendations by Hamel et al. (2020) [19,31], a stop-screening approach was used during title and abstract screening, whereby screening was discontinued once a 95% estimated recall rate was achieved. As a result, not all records remaining after deduplication were screened [19]. Reasons for exclusion exceed the total number of full-texts excluded because each record could be excluded for more than one reason by each reviewer.

Across the nine databases, 10,238 citations were identified. After removing 2556 duplicates, 7682 records remained. Of these, 6469 records were screened at level 1 (title and abstract screening) using a stop-screening approach based on a 95% estimated recall rate. A total of 425 articles were assessed for level 2 (full-text screening), of which 23 studies met the inclusion criteria, and data were extracted (Figure 1) [10].

### 3.2. Characteristics of Sources of Evidence

Of the 23 included studies, 18 (78.3%) were observational [5,6,32,33,34,35,36,37,38,39,40,41,42,43,44,45,46,47]. Five (21.7%) were randomized trials (Table 1) [48,49,50,51,52]. Eight (34.8%) studies were described as pilot/feasibility studies [32,33,34,38,39,41,43,52]. Ten (43.5%) studies described single-institution interventions [6,33,40,42,46,47,51]. Four (17.4%) were multi-institutional [32,35,37,41]. Eight (34.8%) were community-based [36,38,39,43,45,48,49,50]. One (4.3%) was society-based [44].

All studies were conducted in North America, with 21 (91.3%) studies from the USA [5,6,32,34,36,37,38,39,40,41,42,43,44,45,46,47,48,49,50,51,52]. Two studies (8.7%) were from Canada [33,35].

Ten (43.5%) studies specified the types of cancer for which they utilized patient navigation to identify clinical trials [32,34,40,44,46,48,49,50,51,52]. Eight (34.8%) primarily focused on identifying clinical trials for one to three cancer types [32,34,40,46,48,49,50,52]. Two (8.7%) focused on more than three cancer types [44,51]. The most common cancer type was breast cancer, which was included in 8 (34.8%) studies [34,40,46,48,49,50,51,52]. Among the remaining 13 studies that did not specify the cancer type, 8 (34.8%) used patient navigation for clinical trials involving all cancer types [5,6,33,35,37,42,45,47]. Five (21.7%) did not specify any cancer types [36,38,39,41,43].

Fifteen (65.2%) studies utilized patient navigation to increase clinical trial access for specific equity-deserving groups [5,6,34,36,38,39,40,43,45,46,48,49,50,51,52]. Thirteen (56.5%) of those focused on racial, ethnic and Indigenous groups. Studies that included specific racial, ethnic and Indigenous groups focused on Chinese [34,38], American Indian [5], Latinx/Hispanics [36,43,52], African Americans/Blacks [6,39,40,43,46,49], and Filipino [48]. One (4.3%) study focused on low-income and minority patients [45]. One (4.3%) study focused on low English proficiency, ethnically diverse and low-income patients [50].

In 12 (52.2%) studies, navigators directed patients exclusively to internal trials within their center (i.e., institution-driven) [5,6,32,33,40,42,46,47,48,49,51,52]. In contrast, in four (17.4%) studies, navigators directed patients to both internal trials within their center and external trials at other centers (i.e., patient-driven) [35,37,44,50]. Seven (30.4%) studies did not specify whether patients were being matched to internal or external trials [34,36,38,39,41,43,45]. Institution-oriented models were typically described in studies based within a single cancer centre or institutional clinical trial program, whereas patient-oriented models were described in studies that supported identification of both internal and external trial options. Because enrollment outcomes were reported using different denominators and study designs, the available data did not allow a direct comparison of enrollment performance between these models.

### 3.3. Characteristics of Navigation Interventions

Eleven (47.8%) studies described the theoretical underpinnings of the navigation intervention (Table 2) [5,6,32,34,36,38,39,41,43,50,52]. These included the Chronic Care Model [32], Community Health Advisors Network Model [6], systems theory [50], social cognitive theory [52], stages of change model [52], Rogers’ Diffusion of Innovations theory [41] and community engaged formative research/community-based participatory research [5,34,36,38,39,43].

**Figure 2:**
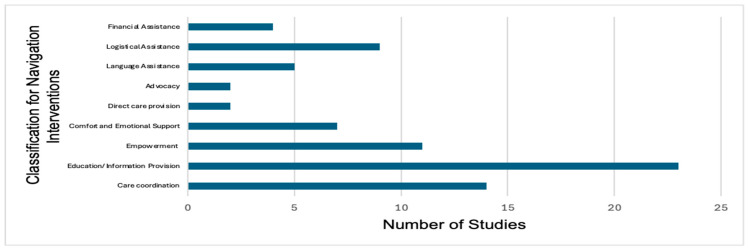
Types of Navigation Services. Note: Categories were based on Chan et al.’s navigation domains: care coordination, education/information provision, empowerment, comfort/emotional support, direct care provision, advocacy, language assistance, logistics assistance, and financial assistance [4].

The most commonly used navigation interventions included education/information provision used by all 23 (100%) included studies [5,6,32,33,34,35,36,37,38,39,40,41,42,43,44,45,46,47,48,49,50,51,52]. Care coordination was used by 14 (60.9%) studies [5,6,32,33,34,35,37,42,44,46,47,48,51,52]. That was followed by empowerment used by 11 (47.8%) studies (Figure 2, Table 2) [6,32,34,38,42,44,45,46,50,51,52]. The navigation interventions that were used the least by two (8.7%) studies each were direct care provision [45] and advocacy [41,46].

Reporting of implementation-relevant elements varied across studies. Elements aligned with TIDieR, such as navigator type, timing of navigation, and intervention components, were commonly reported and are summarized in Table 2 and Table 3. In contrast, elements aligned with RE-AIM and PRISM, including adoption setting, intervention reach, implementation context, maintenance, sustainability, and organizational factors influencing delivery, were less consistently reported. Few studies described long-term integration of navigation into routine clinical trial workflows, and most provided limited information on resources required, institutional supports, or sustainability beyond the study period.

### 3.4. Navigator Training and Certification

Seventeen (73.9%) studies used lay navigators [5,6,32,34,35,36,38,39,40,41,43,45,47,48,49,50,51]. Four (17.4%) studies used nurse navigators (Table 3) [42,44,46,52]. One study (4.3%) did not specify the type of navigator used [37]. One (4.3%) study used a Research Navigator (i.e., a clinical research professional) [33].

The pre-qualifications required for navigators included either formal education or clinical licensure in seven (30.4%) studies [32,35,40,42,44,45,46]. Thirteen (56.5%) navigation programs required community, language, or lived experience [5,6,34,36,37,38,39,41,43,48,49,50,52]. Three (13.0%) studies did not specify any specific pre-qualifications [33,47,51].

Fifteen (65.2%) studies provided the details of training that navigators underwent. Of those studies, ten (43.5%) studies conducted training sessions [32,34,35,36,38,39,41,43,45,51]. Four (17.4%) studies used practical training (e.g., practice sessions, interviewing skills) [6,32,34,40]. Two (8.7%) studies used on-the-job training (e.g., shadowing) [32,50]. Three (13.0%) studies had training that was ongoing [32,35,44]. One (4.3%) study specified that the training consisted of self-paced learning [49].

Eight (34.8%) studies evaluated navigators at the end of training [32,36,38,39,41,42,43,49]. Evaluations consisted of using a performance checklist [32], being assessed through a teach-back session [39], post-training evaluation survey [41], delivering a practice session [36,38,43], using metrics that were compared to baseline numbers (e.g., number of referrals to trials) [42], as well as a post-workshop survey [49].

Two (8.7%) studies mentioned providing certification for navigators [32,49]. Navigators became certified after completing workshops and passing their respective knowledge examination [49] or by completing an offered training course [32].

### 3.5. Outcomes

Thirteen (56.5%) studies evaluated clinical trial enrollment as one of their outcomes [5,6,32,33,34,35,37,40,44,45,46,47,51].

One randomized trial compared enrollment with and without navigation as a primary endpoint and showed no improvement with navigation [51]. Five observational studies compared enrollment with and without navigation [33,34,40,45,47]. Two of those showed no significant improvement [33,34]. Three of those showed significant improvement [40,45,47].

The remaining seven studies that reported enrollment rates with navigation were observational and did not include a comparison group without navigation [5,6,32,35,37,44,46]. Holmes et al. reported 86% enrollment in a clinical trial with navigation among patients who were eligible for a clinical trial, and that the overall accrual of Black patients with newly diagnosed breast cancer increased from 3% to 7% over the study period [46]. Hossami et al. and Sae-Hau et al. navigated all patients interested in clinical trials to internal and external clinical trials and reported enrollment rates of 18.1% and 22.5%, respectively [35,44]. Guadagnolo et al. navigated all patients interested in clinical trials to internal clinical trials and reported an enrollment rate of 22% [5]. Cartmell et al. included only patients eligible for a clinical trial and reported enrollment rates of 76% [32]. Fouad et al. reported an increase in African Americans participating in clinical trials from 9% to 16% over the study period when navigation was provided [6]. Moffitt et al. only reported that there was increased clinical trial enrollment with patient navigation but did not specify further [37].

Secondary outcomes were grouped into five categories: referral and trial-matching outcomes [42], intention or willingness to participate [36,39,43,50,52], knowledge and attitudes [32,34,37,38,39,41,43,50,51,52], satisfaction with navigation [32,33,34], and downstream participation outcomes [44,46,50]. Referral and trial-matching outcomes included the identification of potentially relevant trials, contact with trial sites, referral to trials, and reasons for nonenrollment. Intention and willingness outcomes captured participants’ readiness, self-efficacy, or stated willingness to consider future trial participation. Knowledge and attitude outcomes included understanding of clinical trials, perceived benefits and barriers, trust in medical researchers, and attitudes toward participation. Satisfaction outcomes included patient, navigator, or healthcare professional satisfaction with the navigation intervention. Downstream participation outcomes included enrollment, trial completion, adherence, and barriers encountered after referral. Across studies, these outcomes were reported inconsistently, with variable definitions and denominators, limiting comparison across interventions.

## 4. Discussion

This scoping review represents an up to date, comprehensive synthesis of the evidence on patient navigation specifically for cancer clinical trials. Our findings reveal substantial heterogeneity in program design, implementation, and outcomes, with most studies being observational, single-center initiatives from the United States. While two-thirds focused on racial, ethnic, and Indigenous populations, there was limited attention to other equity-deserving characteristics, intersectionality, or populations outside North America. The predominant use of lay navigators without standardized training or certification highlights a critical gap in professional standards for this evolving field.

Despite navigation’s theoretical appeal and proven effectiveness in cancer screening and treatment, we found limited comparative evidence for its impact on clinical trial enrollment. Most studies reported enrollment rates without comparison groups, and those with comparisons showed inconsistent results, some demonstrating significant benefit, others showing no effect. This heterogeneity may reflect differences in navigator qualifications, intervention intensity, target populations, trial types, or healthcare settings. Concerningly, half of included studies lacked guiding theoretical frameworks, and most failed to use evidence-based outcome reporting frameworks, making it unclear which navigation components are most effective [53]. Most studies described time-limited, grant-funded initiatives with minimal information on long-term implementation. The outcome measurements were inconsistent, only one study followed patients across the full clinical trial continuum (i.e., enrollment through completion), and none of the studies selected conducted a cost–benefit analysis. This limits assessment of the resources, staffing, infrastructure, and long-term sustainability required to implement patient navigation, as it may be a labor-intensive strategy for improving cancer clinical trial access. A study by Ghebre et al. (2014) [54] discussed that a standard approach should be used when evaluating and reporting patient navigation outcomes, and made recommendations for key outcome measures when evaluating and reporting patient navigation outcomes. (e.g., proportion enrolled in a trial, proportion completing the clinical trial) [54].

A critical finding was the predominance of institution-oriented navigation models, in which patients were directed exclusively to trials available at their own centre, rather than patient-oriented cross-institutional models that facilitated access to both internal and external trials. This suggests that many programs were designed around local trial availability and institutional referral pathways. Such models may be practical for centres with active clinical trial portfolios and established research infrastructure, but they may be less applicable for patients with rare cancers, biomarker-defined eligibility, geographic barriers, or limited access to academic cancer centres [55]. Patient-oriented cross-institutional models may better align with individualized trial matching, particularly when suitable trials are unavailable locally. However, these models were less frequently studied and may require additional infrastructure for inter-institutional referral, communication, and follow-up. Because included studies were heterogeneous and rarely designed to compare these models directly, the relative effectiveness of institution-oriented versus patient-oriented navigation for improving trial enrollment remains uncertain.

Although precision oncology and biomarker-driven trial matching are increasingly relevant to cancer clinical trial access, these issues were not consistently reflected in the included studies. Most interventions focused on education, care coordination, trial awareness, referral, or enrollment, with limited reporting on molecular eligibility, genomic testing, or biomarker-based trial matching. Therefore, precision oncology should be interpreted as an important contextual consideration and evidence gap rather than a direct finding of this review.

The geographic concentration of studies in the United States, with only two Canadian studies and no representation from other countries with universal healthcare systems or different healthcare delivery models, limits the generalizability of findings. This geographic bias is particularly notable given that barriers to clinical trial participation may differ substantially across healthcare contexts. The findings should be interpreted in the context of differences in health system organization, academic clinical trial infrastructure, and professional role recognition across countries. Most included studies were conducted in the United States, where cancer centers and grant-funded programs may have distinct resources and incentives to develop navigation services. The structure and recognition of navigator roles may differ substantially in other health systems. Although nurse navigators are established in some oncology settings, this role may not exist in several countries. Similarly, academic programs and institutional responsibilities for trial identification and referral vary internationally. These differences may affect who can serve as a navigator and whether navigation is directed toward institutional trial accrual or broader patient-centered trial matching. As a result, navigation models developed in North American academic or community settings may not be directly transferable to countries with different workforce structures, funding models, or clinical research infrastructure. Additionally, the predominant focus on single cancer types, particularly breast cancer, raises questions about the applicability of navigation strategies to patients with less common malignancies or those requiring biomarker-driven trial matching across multiple potential disease sites. A similar pattern has been observed in the broader patient navigation literature for cancer screening and treatment, where studies are also heavily concentrated in the United States and most commonly focus on breast and colorectal cancers [7].

Several research priorities emerged for our review. First, rigorously designed comparative effectiveness studies, including pragmatic randomized trials, are needed to establish whether navigation increases enrollment equitably across populations. Studies must examine not only enrollment but trial completion, adherence, patient-reported outcomes, and equity impacts. Second, research must identify which navigation components and mechanisms are effective, for whom, and under what conditions. Third, outcomes should expand beyond enrollment to include time to enrollment, informed consent quality, completion rates, patient satisfaction, and whether navigation reduces disparities. Fourth, standardization in navigator training, certification, and scope of practice is essential. While some initiatives have tried to address these issues of standardization for oncology patient navigation more generally, these are often not tailored to unique aspects of cancer clinical trial navigation [56,57]. Professional organizations should collaborate to establish evidence-based competencies and standards, addressing minimum qualifications, core competencies, training curricula, and credentialing processes for cancer clinical trial navigation, similar to standards existing for oncology navigation. Fifth, development and evaluation of patient-driven navigation models that facilitate access across institutions, including partnerships with trial matching platforms and national networks, represents a critical frontier. Such models may be particularly important for rare cancers, biomarker-directed therapies, and patients without access to academic centers.

This scoping review has several limitations. First, only English-language articles were included, which may have excluded relevant studies from countries where patient navigation or similar roles are described using different terminology. Second, although a comprehensive search strategy was used, some navigation programs may exist as local institutional initiatives, quality improvement projects, or unpublished reports that are not indexed in bibliographic databases. Third, the included studies were heterogeneous in design, setting, navigator type, intervention components, target populations, and reported outcomes, which limited direct comparison across studies. This scoping review should be interpreted as a map of the current evidence rather than as a definitive assessment of intervention effectiveness. Therefore, although several studies reported favorable outcomes after navigation, causal conclusions about whether patient navigation improves clinical trial enrollment, which components are most beneficial, or which populations benefit most cannot be drawn from the current evidence. Finally, terminology related to patient navigation, care coordination, case management, and clinical trial support varied across studies, which may have affected study identification and classification despite the use of predefined definitions.

Despite these limitations, this review provides a foundation for future research, practice, and policy by mapping the current landscape of patient navigation for cancer clinical trial access. Patient navigation holds promise for addressing disparities in trial participation, but realizing this potential will require rigorous evaluation, clearer reporting standards, and adaptation to the workforce structures and health system contexts in which navigation programs are implemented.

## 5. Conclusions

Patient navigation is a promising strategy to support access to cancer clinical trials, particularly for populations that experience persistent barriers to trial participation. In this scoping review, we found that existing navigation programs are heterogeneous in their design, navigator roles, training requirements, target populations, and reported outcomes. Although several studies suggested improvements in trial awareness, referral, enrollment, or representation of equity-deserving groups, the evidence remains limited by predominantly observational designs, inconsistent outcome reporting, and a lack of standardized approaches to navigator training and implementation. These findings highlight the need for more rigorous, comparative studies that evaluate not only enrollment, but also trial completion, patient experience, equity impacts, and sustainability. Future research and policy efforts should support standardized reporting, clearer navigator competencies, and patient-centered navigation models that help connect patients with appropriate trials across institutions. Overall, this review maps the current evidence base and identifies key gaps that must be addressed before patient navigation can be fully implemented as an equitable strategy to improve cancer clinical trial access.

## Figures and Tables

**Table 1:** Study Characteristics.

Study (Year)	Country & Setting	Design & Timing	Intervention Aim	Population (N & Inclusion)	Trial Type (Internal/External)	Main Findings
**Blumenthal (1995) [40]**	USA; single institution	Quasi-experimental; timing not reported	Lay navigation for low-income, Black women in breast and cervical screening trial	N = 163; low-income Black women; ≥18; no cancer history	Internal only	Enrollment 20% without navigation vs. 48% with navigation
**Maxwell (2005) [48]**	USA; community-based	RCT; 1998–2000	Lay navigation to recruit Filipino–Americans to breast and cervical cancer screening trial	N = 530; women ≥40; Filipino-American; attended participating churches/organizations	Internal only	84% recruited to attend educational session as part of the trial
**Moffitt (2010) [37]**	USA; multi-institution	Retrospective cohort; 2006–2008	Navigation via online trial registry (FloridaCancerTrials.com)	N = 6350; registry users	Internal + external	Navigation ↑ trial awareness but not trial enrollment; navigated patients 4× more likely to contact matched trial site
**Guadagnolo (2011) [5]**	USA; single institution	Prospective cohort with historical control; 2004–2009	Lay navigation utilization & trial enrollment among American Indian patients	N = 332; any cancer; American Indian	Internal only	Median 12 contacts with navigator per patient; 22% enrolled in a trial
**Michaels (2012) [41]**	USA; multi-institution	Prospective cohort pilot; 2006–2008	Train community leaders, primary care providers, & clinical researchers about trials to increase awareness of & participation	N = 4552 community leaders; N = 549 primary care providers; N = 61 clinical researchers	Not specified	↑ trial knowledge among trained community leaders, primary care providers and clinical researchers; ↑ patient inquiries about trials via websites associated with the initiative
**Holmes (2012) [46]**	USA; single institution	Prospective cohort; 2007–2008	Nurse navigation for trial recruitment for Black breast cancer patients	N = 132; newly diagnosed breast cancer; Black patients	Internal only	86% of eligible enrolled; accrual of Black pts ↑ from 3%→7%; main barrier = lack of trials
**Fracasso (2013) [51]**	USA; single institution	RCT; 2004–2005	6-month lay navigator coaching	N = 75; minority patients; advanced breast, colorectal, lung, or prostate cancer; ECOG 0–2	Internal only	Trial enrollment 16 (intervention) vs. 13 (control) (*p* = 0.351); no effect on trust/attitudes
**Ma (2014) [38]**	USA; community-based	Quasi-experimental pilot; timing not reported	Lay navigation for Chinese Americans to increase knowledge in trials	N = 247; Chinese ethnicity; active community members	Not specified	15/21 trial knowledge measures improved (*p* < 0.05)
**McClung (2015) [34]**	USA; single institution	Quasi-experimental pilot; 2010–2011	Lay navigation use among Chinese patients to improve trial knowledge and attitudes	N = 28; breast & gynecologic cancer; ≥18; Chinese-speaking; ECOG 1–2	Not specified	Average of 317 min of navigation over 8 sessions; 79% satisfied with the navigator information; 86% satisfied navigator interactions; 46% very satisfied with navigator explanation about clinical trials; enrollment increased from 3 pre-navigation vs. 5 post-navigation; attitudes about clinical trials improved
**Green (2015) [39]**	USA; community-based	Quasi-experimental pilot; 2010	Lay navigation to improve trial knowledge & awareness	N = 125; ≥18; targeted African Americans (all races included)	Not specified	↑ trial knowledge (*p* < 0.001); ↑ willingness to participate (*p* < 0.01); ↑ consent understanding (*p* < 0.05)
**Cartmell (2016) [32]**	USA; multi-institution	Prospective cohort pilot; 2010–2011	Lay navigation to improve trial knowledge & enrollment	N = 40; lung & esophageal cancer; ≥18; trial-eligible	Internal only	76% enrolled in a trial; 65% completed trial; trial knowledge improved; high satisfaction with navigation
**Fouad (2016) [6]**	USA; single institution	Prospective cohort; 2007–2014	Lay navigation to increase African American trial participation	N = 272; African American; all cancer types, participating in any cancer trial	Internal only	Trial completion 74.5% vs. 37.5% (*p* = 0.001); 5× more likely to complete trial; participation ↑ from 9%→16%
**Chalela (2018) [52]**	USA; single institution	RCT pilot; timing not reported	Nurse navigation to improve trial understanding, decision readiness & trial consideration for Latina breast cancer patients	N = 77; breast cancer; Latinas; ≥18; trial-eligible	Internal only	↑ trial knowledge (*p* = 0.033); ↑ trial consideration (*p* = 0.008); ↑ decision readiness (*p* < 0.002)
**Williams (2018) [49]**	USA; community-based	RCT; timing not reported	Church-based lay navigation with pastor support for a community-based breast, prostate and colorectal cancer early detection trial	N = 26 lay navigators; African American; ≥21; church members; capable of leading workshops about the trial	Internal only	Pastor attendance associated with ↑ member enrollment (*p* < 0.05)
**Rangel (2019) [36]**	USA; community-based	Quasi-experimental; timing not reported	Lay navigation on trials participation for Hispanics	N = 101; adult Hispanics; Houston, Texas	Not specified	↑ perceived benefits & norms for trial participation; ↓ perceived barriers to trial participation; ↑ self-efficacy & intentions to participate in a trial
**Nickell (2019) [50]**	USA; community-based	RCT; timing not reported	Lay navigation to increase access to trial participation via BreastCancerTrials.org	N = 133; breast cancer patients & survivors; Shanti clients (low English proficiency, ethnically diverse & low-income)	Internal + external	No significant difference in information seeking between navigated and non-navigated patients; ↑ knowledge in some domains; cited barriers to seeking health research information: time, moving on from cancer diagnosis, language barriers
**Cunningham-Erves (2021) [43]**	USA; community-based	Quasi-experimental pilot; 2020–2022	Culturally-appropriate lay navigation to increase knowledge, trust in medical researchers, and intent for trial participation among African Americans & Latinos	N = 198; ≥18; African American/Latino; English/Spanish	Not specified	↑ knowledge (*p* < 0.001); ↑ trust (*p* < 0.001); ↑ willingness to participate in trials (*p* = 0.003)
**Sae-Hau (2021) [44]**	USA; society-based	Retrospective cohort; 2017–2019	Nurse navigation support through The Leukemia & Lymphoma Society’s Clinical Trial Support Center	N = 906; hematologic cancers	Internal + external	Enrollment 16.1% overall among US patients with hematologic cancer; 22.5% if trial search done; main barriers to non-enrollment were standard of care preference or death
**Patel (2021) [45]**	USA; community-based	Quasi-experimental; 2016–2017	Lay navigation for low-income/minority cancer patients	N = 66; Unite Here Health members; ≥18; newly diagnosed cancer	Not specified	↑ trial participation (72% vs. 22%); ↑ quality of life
**Borno (2021) [47]**	USA; single institution	Quasi-experimental; 2016–2020	Lay navigation focused on financial logistics for participation in trials	N = 3470; cancer patients considering trials	Internal only	↑ in trial accrual; ≈1 extra patient/month with navigation; stronger effect in late-phase trials
**Black (2022) [33]**	Canada; single institution	Quasi-experimental pilot; 2018	Research navigator-led kiosk to improve trial accrual	N = 386; ≥18; solid tumors	Internal only	Trial accrual pre-kiosk = 44 vs. post-kiosk = 37; kiosk rated useful & understandable
**Spira (2023) [42]**	USA; single institution	Prospective cohort; 2021–2023	Nurse navigator to assist with trial referrals	N = ~2000; cancer patients	Internal only	↑ in trial referrals; majority of providers satisfied with navigation assistance
**Hossami (2024) [35]**	Canada; multi-institution	Prospective cohort; 2019–2024	National lay navigator program to improve trial accrual	N = 445; Canadians living with cancer	Internal + external	88% had trial identified (median: 1 trial/patient); 27.5% referred to a trial; 18.1% enrolled

Abbreviations: N, number of participants; RCT, randomized controlled trial; ECOG, Eastern Cooperative Oncology Group.

**Table 2:** Description of Patient Navigation Interventions.

Study (Year)	Development of Navigation Intervention	Timing of Navigation Delivery [2]	Care Coordination ^1^	Education/ Information Provision ^2^	Empowerment ^3^	Comfort/ Emotional Support ^4^	Direct Care Provision ^5^	Advocacy ^6^	Language Assistance ^7^	Logistics Assistance ^8^	Financial Assistance ^9^
**Blumenthal (1995) [40]**	-	Community members	-	Educational material provided directly	-	-	-	-	-	-	-
**Maxwell (2005) [48]**	-	Community members	Assisted in locating women who had moved for trial activities	Clinical trial education	-	-	-	-	“Taglish” presentations	Community event participation	-
**Moffitt (2010) [37]**	-	Any time after cancer diagnosis	Trial matching & referral	Educational materials on trials	-	-	-	-	-	Troubleshooting logistics for trial participation	-
**Guadagnolo (2011) [5]**	Community-based participatory research	Any time after cancer diagnosis	Coordinated appointments, follow-up tests, obtained medications/devices	Cancer education	-	Psychosocial support	-	-	Education translated in Lakota	Transportation, lodging	Insurance assistance
**Michaels (2012) [41]**	Rogers’ Diffusion of Innovations theory	Community members	-	Opinion leader training & symposia for community on trials	-	-	-	Advocacy for insurance coverage; simplified consent forms	-	-	-
**Holmes (2012) [46]**	-	Across trial decision-making continuum	Coordinated appointments, medical record handling	Education about disease state and trials	Problem solving	Emotional support (listening, responding)	Performed trial-related clinical services (medications, exams, blood draws)	Advocacy throughout healthcare system	-	Transportation assistance	-
**Fracasso (2013) [51]**	-	At initial clinic visit	Referred to community programs & activities	Trial education	Stage-based counseling (pros/cons of enrollment)	Social support for life issues	-	-	-	-	-
**Ma (2014) [38]**	Community-based participatory research	Community members	-	Group education sessions	Activities/materials to increase self-efficacy	-	-	-	Chinese language handouts	Assistance clarifying information	-
**McClung (2015) [34]**	Community-engaged formative research	Any time after cancer diagnosis	Administrative assistance; referrals	Clinical trial education	Cancer prevention & nutrition support	Caregiver emotional support		-	Translation services	Transportation support	Insurance/financial assistance
**Green (2015) [39]**	Community-based participatory research	Community members	-	Group education sessions	-	-	-	-	-	-	-
**Cartmell (2016) [32]**	Chronic Care Model	Trial discussion, decision, participation	Patient reminders to enhance protocol compliance	17 min educational video; answered trial questions	Screened for logistical barriers	Emotional support (direct or referral)	-	-	-	-	-
**Fouad (2016) [6]**	Community Health Advisors Network Model	Across trial decision-making continuum	Reminder calls; linked to community services; liaised with clinic nurses/social workers	Trial education	Encouraged symptom reporting	Culturally appropriate peer support	-	-	-	Transportation, lodging; social worker referrals	-
**Chalela (2018) [52]**	Social cognitive theory & stages of change model	Initial clinic visit	Scheduled appointments; facilitated medical team communication	Trial information (types, randomization, consent, rationale)	Enhanced self-efficacy in decision-making	Psychosocial support	-	-	Translation support	Addressed participation barriers	-
**Williams (2018) [49]**	-	Community members	-	3-part workshop series on early detection & trials	-	-	-	-	-	-	-
**Rangel (2019) [36]**	Community-based participatory research	Community members	-	Educational material on clinical trials & biobanking	-	-	-	-	-	-	-
**Nickell (2019) [50]**	Systems theory; ‘relational culture’ & ‘health empowerment	After active treatment completed	-	Education about breast cancer research not tied to enrollment in a specific trial	Built resources for self-advocacy	-	-	-	-	-	-
**Cunningham-Erves (2021) [43]**	Community-engaged formative research	Community members	-	Education on clinical trials & biobanking	-	-	-	-	-	-	-
**Sae-Hau (2021) [44]**	-	Any time after cancer diagnosis	Ensured medical record availability; trial search & matching; connected patients to sites	Education about trials	Decision support; psychosocial barrier inquiry	-	-	-	-	Solved participation barriers	Linked patients to financial/travel support programs; assisted insurance appeals
**Patel (2021) [45]**	-	At cancer diagnosis	-	Basic trial education	Goals of care planning, surrogate identification, advance directives	-	Symptom monitoring & management	-	-	-	-
**Borno (2021) [47]**	-	Across trial decision-making continuum	Navigator was primary financial contact	Disseminated financial program info to patients & providers	-	-	-	-	-	-	Supported reimbursement claims (receipts, travel logs, eligibility procedures)
**Black (2022) [33]**	-	Assessment of trial availability	Contacted patients/oncologists with trial options	Provided information about clinical trials	-	-	-	-	-	-	-
**Spira (2023) [42]**	-	Any time after cancer diagnosis	Personalized assistance via routine clinic visits	Addressed trial participation questions; assisted providers with eligibility	Recruitment planning; process gap identification	-	-	-	-	-	-
**Hossami (2024) [35]**	-	Any time after cancer diagnosis	Matching with available trials	Information on available trials	-	-	-	-	-	-	-

Columns 4–12 organized based on Chan et al.’s navigation domains: Care coordination, education/information provision, empowerment, comfort/emotional support, direct care provision, advocacy, language assistance, logistics assistance, and financial assistance [4]. ^1^ **Care Coordination**—Scheduling, reminders, linking services, liaising with providers, monitoring patients. ^2^ **Education/Information Provision**—Providing tailored education, decision aids, and trial information. ^3^ **Empowerment**—Enhancing self-efficacy, problem solving, peer support, and skills building. ^4^ **Comfort/Emotional Support**—Providing psychosocial, cultural, or spiritual support. ^5^ **Direct Care Provision**—Delivering direct nursing or clinical trial–related services. ^6^ **Advocacy**—Acting on patients’ behalf within the healthcare system or policy structures. ^7^ **Language Assistance**—Translation, interpretation, or culturally appropriate materials. ^8^ **Logistics Assistance**—Transportation, scheduling, childcare, outreach, or other practical support. ^9^ **Financial Assistance**—Subsidizing costs, insurance navigation, or financial reimbursement. - = not reported.

**Table 3:** Navigator Training and Certification.

	Navigator Type	Pre-Qualifications Required?	Details of Training	Evaluation of Participants at the End of Training	Did They Receive Certification?	Maintenance of Certification?
**Blumenthal (1995) [40]**	Lay navigators	Black women from the inner-city target population who had attended some college and had experience in grass roots community organizing around women’s health issues.	Trained by project staff in interviewing skills over a ten-week period, and The National Black Women’s Health Project (NBWHP) trained them to deliver the intervention.	N/A	N/A	N/A
**Maxwell (2005) [48]**	Lay navigators	Female Filipino health educator (mainly physician or nurse)	N/A	N/A	N/A	N/A
**Moffitt (2010) [37]**	Not specified	Navigators were bilingual (Spanish and English)	N/A	N/A	N/A	N/A
**Guadagnolo (2011) [5]**	Lay navigators	Navigators were considered culturally competent staff since they were either members of the American Indian communities or connected with that community	N/A	N/A	N/A	N/A
**Michaels (2012) [41]**	Lay navigators	No formal degree or license stated. All navigators were influential community members, primary care providers, or research staff recruited by partnerships or part of the partnership previously.	Training-of-the-trainer session to prepare trainers from each community organization to deliver each module.	Post-training evaluation surveys were conducted	N/A	N/A
**Holmes (2012) [46]**	Nurse navigators	Hired experienced oncology nurse who had appropriate clinical knowledge and sensibility to develop trusting relationships with patients	N/A	N/A	N/A	N/A
**Fracasso (2013) [51]**	Lay navigators	N/A	Didactic training in oncology from oncology-focused meetings such as the Multidisciplinary Cancer Conference, Breast Conference, Colorectal Conference, Genitourinary Conference, Thoracic Oncology Conference, and Clinical Research Associate Forum.	N/A	N/A	N/A
**Ma (2014) [38]**	Lay navigators	Active volunteer Asian community representatives familiar with the population served.	Community health educators received a 12 h training on clinical education	All trained Asian Community Health Educators delivered a practice session of the clinical trial education following training.	N/A	N/A
**McClung (2015) [34]**	Lay navigators	Bilingual Chinese cancer survivors recruited as navigators	Nine-hour training program developed based on results of a focus group and health professional interviews. Topics included: emotional communication skills, role of the navigator, obtaining resources/referrals, confidentiality, barriers to care, cancer basics, clinical trials, navigator documentation, role-plays, questions and answers.	N/A	N/A	N/A
**Green (2015) [39]**	Lay navigators	Lead staff member (executive director or director of programs) selected from each of the following organizations: University of North Carolina at Chapel Hill, Education Network to Advance Cancer Clinical Trials (ENACCT), and four community-based organizations.	Trainers from each community organization underwent two days of sessions to prepare them to deliver each module	Teach back sessions used to assess retention and delivery of content	N/A	N/A
**Cartmell (2016) [32]**	Lay navigators	Range of educational levels (i.e., licensed practical nursing to a non-clinical master’s degree)	Lay navigators participated in a three-part training program that included a 1.5-day didactic session, shadowing experiences, a 1-day practical session and bi-weekly conference calls.	A performance checklist was used to evaluate the navigator’s mastery of navigation skills in cultural competencies.	Each navigator completed the University of Miami’s Basic Citi Course Training for Human Subjects Research and the Education Network to Advance Cancer Clinical Trials (ENACCT) Foundation’s Training Course.	N/A
**Fouad (2016) [6]**	Lay navigators	Individuals already serving as community health advisors for cancer prevention and control or embodied qualities of community health advisors. Matched demographic characteristics of the patients.	Training curriculum and training manual developed. Training consisted of a background section and three modules. Module I was an overview of the concept of patient navigation, and training about cancer clinical trials using NCI Cancer Clinical Trials publications. Module II was about the navigation process, interacting with patients, patient interventions, and the clinic environment. Module III consisted of case-management and data-management training, along with instructions on how to explain the clinical trials consent process to potential participants.	N/A	N/A	N/A
**Chalela (2018) [52]**	Nurse navigators	Nurse navigator was female, bicultural, and bilingual	N/A	N/A	N/A	N/A
**Williams (2018) [49]**	Lay navigators	The pastor identified one man and one woman to serve as church community health advisor. Must be older than 21 years, self-identification as African American, regular attendance at enrolled church, able to complete Project HEAL (Health through Early Awareness and Learning), training, regular internet access and comfortability with Web-based activities, able to recruit 30 participants to workshop, and able to lead 3-part workshop series.	Community health advisors were trained by HEAL staff to conduct educational workshop series regarding early detection. Community health advisors in the Technology churches completed the same training and certification using a self-paced Web-based portal, independently.	Post-workshop survey after community health advisors delivered the 3-part workshop series allowing evaluation of their experiences in leading the workshops.	Knowledge examination administered to community health advisors prior to workshop implementation to become certified.	N/A
**Rangel (2019) [36]**	Lay navigators	Experienced promotoras with prior community health education work; no formal educational degree requirement specified	In-person training for each curriculum lasted approximately 2 h each	Promotoras completed practice sessions to ensure content knowledge and comfortability with the group format delivery	N/A	N/A
**Nickell (2019) [50]**	Lay navigators	Shanti Care Navigators; speak with clients in English, Spanish, Cantonese, and Mandarin and provide services in non-clinical setting	Navigators are trained through the Shanti Model of Peer Support™, which is a non-directive, client-centred mode of communication focusing on the skills of active-listening, harm-reduction, and compassionate presence	N/A	N/A	N/A
**Cunningham-Erves (2021) [43]**	Lay navigators	Embedded community members located throughout Middle Tennessee with trusted relationships throughout the community	Trained or re-trained six community health educators in a half day training	Training evaluation included trainee practice sessions. The trainee had to demonstrate mastery in the following four areas: understanding of cancer disease and how to prevent it; defining clinical trials and their process; describing the benefits and risks of clinical trial participation; and discussing the process of clinical trial enrollment and participant protections. Feedback was provided.	N/A	N/A
**Sae-Hau (2021) [44]**	Nurse navigators	Oncology nurses including advanced practice nurses, nurse practitioners (pediatric and adult), research nurses, and nurse educators	Ongoing education in hematologic malignancy physiology, treatment methods, stem-cell transplantation, clinical trials, and genomics.	N/A	N/A	N/A
**Patel (2021) [45]**	Lay navigators	Two full-time equivalent Health Advocates that are members of the local community with a college degree under the supervision of a nurse.	Trained Health Advocates in didactic and skills-based training over 2-day in-person and 1 half-day virtual sessions supplemented with weekly 1 h virtual sessions during the first month of the initiative. Focused on basics of cancer, social determinants of health and its impact on cancer outcome disparities, advance care planning, symptom assessment, and importance of and challenges in clinical trial participation among historically under-represented populations.	N/A	N/A	N/A
**Borno (2021) [47]**	Lay navigator	N/A	N/A	N/A	N/A	N/A
**Black (2022) [33]**	Research navigators	N/A	N/A	N/A	N/A	N/A
**Spira (2023) [42]**	Nurse navigators	Oncology nurse with 20 years of experience	N/A	Specific metrics will be developed (e.g., overall site accrual, number of referrals to trials, and increases in enrollment by ‘low enroller’ providers) and compared to baseline numbers to measure effectiveness of the clinical trials navigator position.	N/A	N/A
**Hossami (2024) [35]**	Lay navigators	Clinical trials navigators required a university degree or were placement students coming from master’s programs or clinical trials management courses. They were required to also have a proficiency in reading scientific literature, as this is required to interpret the clinicaltrials.gov website.	Navigators underwent onboarding program led by team staff, along with weekly quality improvement sessions regarding their clinical trials searches.	N/A	N/A	N/A

## Data Availability

No new data were created or analyzed in this study. Data sharing is not applicable to this article.

## Supplementary Materials

The following supporting information can be downloaded at: https://www.mdpi.com/article/10.3390/curroncol33060341/s1, Table S1: Search Strategy; Table S2: PRISMA_2020_Checklist.
