# Supplementary material for: Connecting Patients with Clinical Trials Using Patient Navigation: A Scoping Review
Source: Curr Oncol. 2026 Jun 8;33(6):341. doi: 10.3390/curroncol33060341 (PMC13298245; doi:10.3390/curroncol33060341)
Supplement: Supplementary file 1 [file curroncol-33-00341-s001.zip › curroncol-4285108-Table S1 Search Strategy.pdf]

Table S1: Search Strategy

Initial Search Strategy

Ovid MEDLINE(R) and Epub Ahead of Print, In-Process, In-Data-Review & Other Non-Indexed Citations and Daily <1946 to December 20, 2023>		
Search	Querty	Results
1	therapeutic human experimentation/	1803
2	exp clinical trials as topic/mt, og, st or clinical trial protocols as topic/mt, og, st	34853
3	(exp clinical trials as topic/ or clinical trial protocols as topic/) and (research design/ or patient selection/ or patient satisfaction/ or patient participation/ or "patient acceptance of health care"/ or research subjects/ or human experimentation/ or patient education as topic/ or stakeholder participation/ or health services accessibility/)	41205
4	((clinical research or trial or trials) adj2 (coordinat* or co-ordinat* or recruiter* or recruitment or recruiting or nurs* or advoca* or participation or facilitat* or engag* or accrual* or enrol?ment or selection or educat* or underrepresent* or under represent* or access*)).ti,ab,kf.	12864
5	((research or trial or trials) adj5 ((participant* or subject or subjects or patient*) adj2 (recruitment or accrual* or enrol?ment or selection or barrier* or facilitat*))).ti,ab,kf.	4016
6	or/1-5	78105
7	patient navigation/ or patient-centered care/ or case management/ or case managers/ or community health workers/ or patient advocacy/	64985
8	(navigator* or case manage* or care manage* or key worker* or link worker* or linkworker* or keyworker* or promotora* or community matron* or guided care or CHN* or LHW* or lay health worker* or community health worker* or village health worker* or outreach worker*).ti,ab,kf.	46459
9	((coordinator* or co-ordinator* or facilitator* or coach* or advisor or advisors or advocate* or navigat* or liaison* or broker* or educator* or counselor* or counsellor*) adj3 (care or case or health or healthcare or patient* or client* or family or families or system or systems or service* or peer or peers or community)).ti,ab,kf.	49809
10	or/7-9	145231
11	6 and 10	1417
12	limit 11 to english language	1379
13	(autobiography or biography or comment or editorial or interview or letter or news or newspaper article).pt.	2634853

14	12 not 13	1242

Embase <1974 to 2023 Week 50>		
Search	Query	Results
1	therapeutic research/	424
2	(exp "clinical trial (topic)"/ or clinical trial protocol/) and (methodology/ or patient selection/ or patient satisfaction/ or patient participation/ or patient attitude/ or research subject/ or human experiment/ or patient education/ or stakeholder engagement/ or health care access/ or eligibility/ or eligibility criteria/)	35321
3	((clinical research or trial or trials) adj2 (coordinat* or co-ordinat* or recruiter* or recruitment or recruiting or nurs* or advoca* or participation or facilitat* or engag* or accrual* or enrol?ment or selection or educat* or underrepresent* or under represent* or access*)).ti,ab,kf.	22866
4	((research or trial or trials) adj5 ((participant* or subject or subjects or patient*) adj2 (recruitment or accrual* or enrol?ment or selection or barrier* or facilitat*))).ti,ab,kf.	6557
5	or/1-4	62000
6	case management/ or case manager/ or health auxiliary/ or exp patient advocacy/	49810
7	(navigator* or case manage* or care manage* or key worker* or link worker* or linkworker* or keyworker* or promotora* or community matron* or guided care or CHN* or LHW* or lay health worker* or community health worker* or village health worker* or outreach worker*).ti,ab,kf.	62490
8	((coordinator* or co-ordinator* or facilitator* or coach* or advisor or advisors or advocate* or navigat* or liaison* or broker* or educator* or counselor* or counsellor*) adj3 (care or case or health or healthcare or patient* or client* or family or families or system or systems or service* or peer or peers or community)).ti,ab,kf.	68837
9	or/6-8	156896
10	5 and 9	1514
11	limit 10 to english language	1507
12	(book or chapter or editorial or letter or note).pt.	3122561
13	11 not 12	1439

EBM Reviews - Cochrane Central Register of Controlled Trials <November 2023>		
Search	Query	Results
1	therapeutic human experimentation/	89

2	exp clinical trials as topic/mt, og, st or clinical trial protocols as topic/mt, og, st	0
3	(exp clinical trials as topic/ or clinical trial protocols as topic/) and (research design/ or patient selection/ or patient satisfaction/ or patient participation/ or "patient acceptance of health care"/ or research subjects/ or human experimentation/ or patient education as topic/ or stakeholder participation/ or health services accessibility/)	6889
4	((clinical research or trial or trials) adj2 (coordinat* or co-ordinat* or recruiter* or recruitment or recruiting or nurs* or advoca* or participation or facilitat* or engag* or accrual* or enrol?ment or selection or educat* or underrepresent* or under represent* or access*)).ti,ab,kw.	8212
5	((research or trial or trials) adj5 ((participant* or subject or subjects or patient*) adj2 (recruitment or accrual* or enrol?ment or selection or barrier* or facilitat*))).ti,ab,kw.	3018
6	or/1-5	16960
7	patient navigation/ or patient-centered care/ or case management/ or case managers/ or community health workers/ or patient advocacy/	2704
8	(navigator* or case manage* or care manage* or key worker* or link worker* or linkworker* or keyworker* or promotora* or community matron* or guided care or CHN* or LHW* or lay health worker* or community health worker* or village health worker* or outreach worker*).ti,ab,kw.	8987
9	((coordinator* or co-ordinator* or facilitator* or coach* or advisor or advisors or advocate* or navigat* or liaison* or broker* or educator* or counselor* or counsellor*) adj3 (care or case or health or healthcare or patient* or client* or family or families or system or systems or service* or peer or peers or community)).ti,ab,kw.	17042
10	or/7-9	
11	6 and 10	574
12	limit 11 to english language	565
13	(comment or editorial or letter or note).pt.	27712
14	12 not 13	559

CINAHL via EBSCO		
Search	Query	Results

1	(MH "Clinical Trials+/MT/OG/ST")	8769
2	(MH "Clinical Trials+") and ((MH "study design") or (MH "patient selection") or (MH "patient satisfaction") or (MH "consumer participation") or (MH "research subjects+") or (MH "patient education") or (MH "stakeholder participation") or (MH "health services accessibility"))	34877
3	TI(("clinical research" or trial or trials) N2 (coordinat* or "co ordinat*" or recruiter* or recruitment or recruiting or nurs* or advoca* or participation or facilitat* or engag* or accrual* or enrol#ment or selection or educat* or underrepresent* or "under represent*" or access*)) OR AB(("clinical research" or trial or trials) N2 (coordinat* or "co ordinat*" or recruiter* or recruitment or recruiting or nurs* or advoca* or participation or facilitat* or engag* or accrual* or enrol#ment or selection or educat* or underrepresent* or "under represent*" or access*))	10434
4	TI((research or trial or trials) N5 ((participant* or subject or subjects or patient*) N2 (recruitment or accrual* or enrol#ment or selection or barrier* or facilitat*))) OR AB((research or trial or trials) N5 ((participant* or subject or subjects or patient*) N2 (recruitment or accrual* or enrol#ment or selection or barrier* or facilitat*)))	2271
5	S1 or S2 or S3 or S4	49915
6	(MH "patient navigation") or (MH "patient centered care") or (MH "case management+") or (MH "case managers+") or (MH "community health workers") or (MH "patient advocacy")	75939
7	TI(navigator* or "case manage*" or "care manage*" or "key worker*" or "link worker*" or linkworker* or keyworker* or promotora* or "community matron*" or "guided care" or CHN* or LHW* or "lay health worker*" or "community health worker*" or "village health worker*" or "outreach worker*") or AB(navigator* or "case manage*" or "care manage*" or "key worker*" or "link worker*" or linkworker* or keyworker* or promotora* or "community matron*" or "guided care" or CHN* or LHW* or "lay health worker*" or "community health worker*" or "village health worker*" or "outreach worker	42631
8	TI((coordinator* or "co-ordinator*" or facilitator* or coach* or advisor or advisors or advocate* or navigat* or liaison* or broker* or educator* or counselor* or counsellor*) N3 (care or case or health or healthcare or patient* or client* or family or families or system or systems or service* or peer or peers or community)) OR AB((coordinator* or "co-ordinator*" or facilitator* or coach* or advisor or advisors or advocate* or navigat* or liaison* or broker* or educator* or counselor* or counsellor*) N3 (care or case or health	38159

	or healthcare or patient* or client* or family or families or system or systems or service* or peer or peers or community))	
9	S6 or S7 or S8	125205
10	S5 and S9	1690
11	S10	1676
12	(ZT "biography") or (ZT "book") or (ZT "book chapter") or (ZT "commentary") or (ZT "editorial") or (ZT "interview") or (ZT "letter") or (ZT "newspaper")	851036
13	S11 not S12	1611

Epistemonikos		
Search	Query	Results
1	(title:(trial OR trials OR "clinical research") OR abstract:(trial OR trials OR "clinical research")) AND (title:("research coordinator" OR "research co-ordinator" OR "trial coordinator" OR "trial co-ordinator" OR recruiter* OR recruitment OR "research nurse" OR "trial nurse" OR accrual* OR enrolment OR enrollment OR underrepresent*) OR abstract:("research coordinator" OR "research co-ordinator" OR "trial coordinator" OR "trial co-ordinator" OR recruiter* OR recruitment OR "research nurse" OR "trial nurse" OR accrual* OR enrolment OR enrollment OR underrepresent*)) AND (title:(navigator* OR "case manager" OR "case management" OR "care manager" OR "care management" OR "key worker" OR "link worker" OR linkworker* OR keyworker* OR promotora* OR "community matron" OR "guided care" OR CHN* OR LHW* OR "lay health worker" OR "community health worker" OR "village health worker" OR "outreach worker" OR facilitator* OR coach* OR advisor OR advisors OR advocate* OR navigat* OR liaison* OR broker* OR educator* OR counselor* OR counsellor*) OR abstract:(navigator* OR "case manager" OR "case management" OR "care manager" OR "care management" OR "key worker" OR "link worker" OR linkworker* OR keyworker* OR promotora* OR "community matron" OR "guided care" OR CHN* OR LHW* OR "lay health worker" OR "community health worker" OR "village health worker" OR "outreach worker" OR facilitator* OR coach* OR advisor OR advisors OR advocate* OR navigat* OR liaison* OR broker* OR educator* OR counselor* OR counsellor*))	1868

PROSPERO		
Search	Query	Results
1	MeSH DESCRIPTOR Therapeutic Human Experimentation EXPLODE ALL TREES	3

2	((MeSH DESCRIPTOR clinical trials as topic EXPLODE ALL TREES) or (MeSH DESCRIPTOR clinical trial protocols as topic)) and ((MeSH DESCRIPTOR research design) or (MeSH DESCRIPTOR patient selection) or (MeSH DESCRIPTOR patient satisfaction) or (MeSH DESCRIPTOR patient participation) or (MeSH DESCRIPTOR "patient acceptance of health care") or (MeSH DESCRIPTOR research subjects) or (MeSH DESCRIPTOR human experimentation) or (MeSH DESCRIPTOR patient education as topic) or (MeSH DESCRIPTOR stakeholder participation) or (MeSH DESCRIPTOR health services accessibility))	92
3	(("clinical research" or trial or trials) adj2 (coordinat* or co-ordinat* or recruiter* or recruitment or recruiting or nurs* or advoca* or participation or facilitat* or engag* or accrual* or enrolment or enrollment or selection or educat* or underrepresent* or under represent* or access*))	1547
4	((research or trial or trials) adj5 ((participant* or subject or subjects or patient*) adj2 (recruitment or accrual* or enrol?ment or selection or barrier* or facilitat*)))	179
5	#1 OR #2 OR #3 OR #4	1786
6	(MeSH DESCRIPTOR patient navigation) or (MeSH DESCRIPTOR patient-centered care) or (MeSH DESCRIPTOR case management) or (MeSH DESCRIPTOR case managers) or (MeSH DESCRIPTOR community health workers) or (MeSH DESCRIPTOR patient advocacy)	95
7	navigator* or "case manager" or "case management" or "care manager" or "care management" or "key worker" or "link worker" or linkworker* or keyworker* or promotora* or "community matron" or "guided care" or CHN* or LHW* or "lay health worker" or "community health worker" or "village health worker" or "outreach worker"	1182
8	((coordinator* or co-ordinator* or facilitator* or coach* or advisor or advisors or advocate* or navigat* or liaison* or broker* or educator* or counselor* or counsellor*) adj3 (care or case or health or healthcare or patient* or client* or family or families or system or systems or service* or peer or peers or community))	1639
9	#6 OR #7 OR #8	2710
10	#5 AND #9	31

Trip Pro		
Search	Query	Results
1	(trial OR trials OR "clinical research") AND ("research coordinator" OR "research co ordinator" OR "trial coordinator" OR "trial co ordinator" OR recruiter* OR "subject recruitment" OR "participant recruitment" OR "patient recruitment" OR "research nurse" OR "trial nurse" OR accrual* OR underrepresent*) AND (navigator* OR	1161

	"case manager" OR "case management" OR "care manager" OR "care management" OR promotora* OR "community matron" OR "guided care" OR "lay health worker" OR "community health worker" OR "village health worker" OR "outreach worker" OR facilitator* OR coach* OR advisor OR advisors OR "patient advocate" OR navigation* OR liaison* OR broker* OR educator* OR counselor* OR counsellor*)	

ICTRP (WHO)		
Search	Query	Results
1	("research coordinator" OR "research co ordinator" OR "trial coordinator" OR "trial co ordinator" OR recruiter OR recruitment OR enrollment OR enrolment OR "research nurse" OR "trial nurse" OR accrual OR underrepresented OR underrepresentation) AND (navigator OR "case manager" OR "case management" OR "care manager" OR "care management" OR promotora OR "community matron" OR "guided care" OR "lay health worker" OR "community health worker" OR "village health worker" OR "outreach worker" OR facilitator OR coach OR advisor OR advisors OR "patient advocate" OR navigation OR liaison OR broker OR educator OR counselor OR counsellor)	142

AHRQ via Google		
Search	Query	Results
1	("clinical research" OR trial) AND (coordinator OR recruiter OR recruitment OR underrepresented) AND (navigator OR "case manager" OR "case management" OR "care management" OR "guided care" OR "lay health worker" OR "community health worker" OR coach OR advisor OR advisors OR "patient advocate" OR navigation OR liaison OR broker OR counselor) site:effectivehealthcare.ahrq.gov/products	57

Google Scholar		
Search	Query	Results
1	("clinical research" OR trial) AND (coordinator OR recruiter OR recruitment OR underrepresented) AND (navigator OR "case manager" OR "case management" OR "care management" OR "guided care" OR "lay health worker" OR "community health worker" OR coach OR advisor OR advisors OR "patient advocate" OR navigation OR liaison OR broker OR counselor) - first 10 pages	100

Updated Complete Search Strategy

Ovid MEDLINE(R) Epub Ahead of Print and In-Process, In-Data-Review & Other Non-Indexed Citations and Daily <March 04, 2025>		
Search	Querty	Results
1	therapeutic human experimentation/	1805
2	exp clinical trials as topic/mt, og, st or clinical trial protocols as topic/mt, og, st	35964
3	(exp clinical trials as topic/ or clinical trial protocols as topic/) and (research design/ or patient selection/ or patient satisfaction/ or patient participation/ or "patient acceptance of health care"/ or research subjects/ or human experimentation/ or patient education as topic/ or stakeholder participation/ or health services accessibility/)	43365
4	((clinical research or trial or trials) adj2 (coordinat* or co-ordinat* or recruiter* or recruitment or recruiting or nurs* or advoca* or participation or facilitat* or engag* or accrual* or enrol?ment or selection or educat* or underrepresent* or under represent* or access*)).ti,ab,kf.	14322
5	((research or trial or trials) adj5 ((participant* or subject or subjects or patient*) adj2 (recruitment or accrual* or enrol?ment or selection or barrier* or facilitat*))).ti,ab,kf.	4545
6	or/1-5	82425
7	patient navigation/ or patient-centered care/ or case management/ or case managers/ or community health workers/ or patient advocacy/	67184
8	(navigator* or case manage* or care manage* or key worker* or link worker* or linkworker* or keyworker* or promotora* or community matron* or guided care or CHN* or LHW* or lay health worker* or community health worker* or village health worker* or outreach worker*).ti,ab,kf.	50639
9	((coordinator* or co-ordinator* or facilitator* or coach* or advisor or advisors or advocate* or navigat* or liaison* or broker* or educator* or counselor* or counsellor*) adj3 (care or case or health or healthcare or patient* or client* or family or families or system or systems or service* or peer or peers or community)).ti,ab,kf.	56321
10	or/7-9	156812
11	6 and 10	1551
12	limit 11 to english language	1511
13	(autobiography or biography or comment or editorial or interview or letter or news or newspaper article).pt.	2743423
14	12 not 13	1371
15	limit 14 to yr="2023 -Current"	142

Embase <1974 to 2025 Week 09>		
Search	Query	Results
1	therapeutic research/	460
2	(exp "clinical trial (topic)"/ or clinical trial protocol/) and (methodology/ or patient selection/ or patient satisfaction/ or patient participation/ or patient attitude/ or research subject/ or human experiment/ or patient education/ or stakeholder engagement/ or health care access/ or eligibility/ or eligibility criteria/)	37743
3	((clinical research or trial or trials) adj2 (coordinat* or co-ordinat* or recruiter* or recruitment or recruiting or nurs* or advoca* or participation or facilitat* or engag* or accrual* or enrol?ment or selection or educat* or underrepresent* or under represent* or access*)).ti,ab,kf.	25481
4	((research or trial or trials) adj5 ((participant* or subject or subjects or patient*) adj2 (recruitment or accrual* or enrol?ment or selection or barrier* or facilitat*))).ti,ab,kf.	7340
5	or/1-4	67503
6	case management/ or case manager/ or health auxiliary/ or exp patient advocacy/	53088
7	(navigator* or case manage* or care manage* or key worker* or link worker* or linkworker* or keyworker* or promotora* or community matron* or guided care or CHN* or LHW* or lay health worker* or community health worker* or village health worker* or outreach worker*).ti,ab,kf.	67458
8	((coordinator* or co-ordinator* or facilitator* or coach* or advisor or advisors or advocate* or navigat* or liaison* or broker* or educator* or counselor* or counsellor*) adj3 (care or case or health or healthcare or patient* or client* or family or families or system or systems or service* or peer or peers or community)).ti,ab,kf.	76871
9	or/6-8	170927
10	5 and 9	1753
11	limit 10 to english language	1744
12	(book or chapter or editorial or letter or note).pt.	3245726
13	11 not 12	1664
14	limit 13 to yr="2023 -Current"	385

EBM Reviews - Cochrane Central Register of Controlled Trials <January 2025>		
Search	Query	Results
1	therapeutic human experimentation/	96

2	exp clinical trials as topic/mt, og, st or clinical trial protocols as topic/mt, og, st	1
3	(exp clinical trials as topic/ or clinical trial protocols as topic/) and (research design/ or patient selection/ or patient satisfaction/ or patient participation/ or "patient acceptance of health care"/ or research subjects/ or human experimentation/ or patient education as topic/ or stakeholder participation/ or health services accessibility/)	8574
4	((clinical research or trial or trials) adj2 (coordinat* or co-ordinat* or recruiter* or recruitment or recruiting or nurs* or advoca* or participation or facilitat* or engag* or accrual* or enrol?ment or selection or educat* or underrepresent* or under represent* or access*)).ti,ab,kw.	9018
5	((research or trial or trials) adj5 ((participant* or subject or subjects or patient*) adj2 (recruitment or accrual* or enrol?ment or selection or barrier* or facilitat*))).ti,ab,kw.	3258
6	or/1-5	19487
7	patient navigation/ or patient-centered care/ or case management/ or case managers/ or community health workers/ or patient advocacy/	3041
8	(navigator* or case manage* or care manage* or key worker* or link worker* or linkworker* or keyworker* or promotora* or community matron* or guided care or CHN* or LHW* or lay health worker* or community health worker* or village health worker* or outreach worker*).ti,ab,kw.	8764
9	((coordinator* or co-ordinator* or facilitator* or coach* or advisor or advisors or advocate* or navigat* or liaison* or broker* or educator* or counselor* or counsellor*) adj3 (care or case or health or healthcare or patient* or client* or family or families or system or systems or service* or peer or peers or community)).ti,ab,kw.	9244
10	or/7-9	18871
11	6 and 10	709
12	limit 11 to english language	701
13	(comment or editorial or letter or note).pt.	21003
14	12 not 13	695
15	limit 14 to yr="2023 -Current"	111

CINAHL via EBSCO

Search	Query	Results
1	(MH "Clinical Trials+/MT/OG/ST")	8545
2	(MH "Clinical Trials+") and ((MH "study design") or (MH "patient selection") or (MH "patient satisfaction") or (MH "consumer participation") or (MH "research subjects+") or (MH "patient education") or (MH "stakeholder participation") or (MH "health services accessibility"))	35522
3	TI(("clinical research" or trial or trials) N2 (coordinat* or "co ordinat*" or recruiter* or recruitment or recruiting or nurs* or advoca* or participation or facilitat* or engag* or accrual* or enrol#ment or selection or educat* or underrepresent* or "under represent*" or access*)) OR AB(("clinical research" or trial or trials) N2 (coordinat* or "co ordinat*" or recruiter* or recruitment or recruiting or nurs* or advoca* or participation or facilitat* or engag* or accrual* or enrol#ment or selection or educat* or underrepresent* or "under represent*" or access*))	10736
4	TI((research or trial or trials) N5 ((participant* or subject or subjects or patient*) N2 (recruitment or accrual* or enrol#ment or selection or barrier* or facilitat*))) OR AB((research or trial or trials) N5 ((participant* or subject or subjects or patient*) N2 (recruitment or accrual* or enrol#ment or selection or barrier* or facilitat*)))	2340
5	S1 or S2 or S3 or S4	50689
6	(MH "patient navigation") or (MH "patient centered care") or (MH "case management+") or (MH "case managers+") or (MH "community health workers") or (MH "patient advocacy")	81112
7	TI(navigator* or "case manage*" or "care manage*" or "key worker*" or "link worker*" or linkworker* or keyworker* or promotora* or "community matron*" or "guided care" or CHN* or LHW* or "lay health worker*" or "community health worker*" or "village health worker*" or "outreach worker*") or AB(navigator* or "case manage*" or "care manage*" or "key worker*" or "link worker*" or linkworker* or keyworker* or promotora* or "community matron*" or "guided care" or CHN* or LHW* or "lay health worker*" or "community health worker*" or "village health worker*" or "outreach worker	43638
8	TI((coordinator* or "co-ordinator*" or facilitator* or coach* or advisor or advisors or advocate* or navigat* or liaison* or broker* or educator* or counselor* or counsellor*) N3 (care or case or health or healthcare or patient* or client* or family or families or system or systems or service* or peer or peers or community)) OR AB((coordinator* or "co-ordinator*" or facilitator* or coach* or advisor or advisors or advocate* or navigat* or liaison* or broker* or educator* or counselor* or counsellor*) N3 (care or case or health	39126

	or healthcare or patient* or client* or family or families or system or systems or service* or peer or peers or community))	
9	S6 or S7 or S8	130910
10	S5 and S9	1772
11	S10 Limiters - English Language	1755
12	(ZT "biography") or (ZT "book") or (ZT "book chapter") or (ZT "commentary") or (ZT "editorial") or (ZT "interview") or (ZT "letter") or (ZT "newspaper")	847828
13	S11 not S12	1688
14	S13 Limiters - Publication Date: 20230101-	169

Epistemonikos		
Search	Query	Results
1	(title:(trial OR trials OR "clinical research") OR abstract:(trial OR trials OR "clinical research")) AND (title:("research coordinator" OR "research co-ordinator" OR "trial coordinator" OR "trial co-ordinator" OR recruiter* OR recruitment OR "research nurse" OR "trial nurse" OR accrual* OR enrolment OR enrollment OR underrepresent*) OR abstract:("research coordinator" OR "research co-ordinator" OR "trial coordinator" OR "trial co-ordinator" OR recruiter* OR recruitment OR "research nurse" OR "trial nurse" OR accrual* OR enrolment OR enrollment OR underrepresent*)) AND (title:(navigator* OR "case manager" OR "case management" OR "care manager" OR "care management" OR "key worker" OR "link worker" OR linkworker* OR keyworker* OR promotora* OR "community matron" OR "guided care" OR CHN* OR LHW* OR "lay health worker" OR "community health worker" OR "village health worker" OR "outreach worker" OR facilitator* OR coach* OR advisor OR advisors OR advocate* OR navigat* OR liaison* OR broker* OR educator* OR counselor* OR counsellor*) OR abstract:(navigator* OR "case manager" OR "case management" OR "care manager" OR "care management" OR "key worker" OR "link worker" OR linkworker* OR keyworker* OR promotora* OR "community matron" OR "guided care" OR CHN* OR LHW* OR "lay health worker" OR "community health worker" OR "village health worker" OR "outreach worker" OR facilitator* OR coach* OR advisor OR advisors OR advocate* OR navigat* OR liaison* OR broker* OR educator* OR counselor* OR counsellor*)), filtered to publication year 2023-2025	710

PROSPERO		
Search	Query	Results
1	MeSH DESCRIPTOR Therapeutic Human Experimentation EXPLODE ALL TREES	6

2	((MeSH DESCRIPTOR clinical trials as topic EXPLODE ALL TREES) or (MeSH DESCRIPTOR clinical trial protocols as topic)) and ((MeSH DESCRIPTOR research design) or (MeSH DESCRIPTOR patient selection) or (MeSH DESCRIPTOR patient satisfaction) or (MeSH DESCRIPTOR patient participation) or (MeSH DESCRIPTOR "patient acceptance of health care") or (MeSH DESCRIPTOR research subjects) or (MeSH DESCRIPTOR human experimentation) or (MeSH DESCRIPTOR patient education as topic) or (MeSH DESCRIPTOR stakeholder participation) or (MeSH DESCRIPTOR health services accessibility))	17037
3	((research OR trial OR trials) ADJ2 (coordinat* OR recruiter* OR recruitment OR recruiting OR nurse OR advoca* OR participation OR facilitat* OR engag* OR accrual* OR enrolment OR enrollment OR selection OR underrepresent* OR represented OR access*))	2100
4	((participant* OR subject OR subjects OR patient*) ADJ2 (recruitment OR accrual* OR enrolment OR enrollment OR selection OR barrier* OR facilitat*))	10671
5	#1 OR #2 OR #3 OR #4	29192
6	(MeSH DESCRIPTOR patient navigation) or (MeSH DESCRIPTOR patient-centered care) or (MeSH DESCRIPTOR case management) or (MeSH DESCRIPTOR case managers) or (MeSH DESCRIPTOR community health workers) or (MeSH DESCRIPTOR patient advocacy)	445
7	navigator* or "case manager" or "case management" or "care manager" or "care management" or "key worker" or "link worker" or linkworker* or keyworker* or promotora* or "community matron" or "guided care" or CHN* or LHW* or "lay health worker" or "community health worker" or "village health worker" or "outreach worker"	1481
8	((coordinator* OR ordinator* OR facilitator* OR coach* OR advisor OR advisors OR advocate* OR navigat* OR liaison* OR broker* OR educator* OR counselor* OR counsellor*) ADJ3 (care OR case OR health OR healthcare OR patient* OR client* OR family OR families OR system OR systems OR service* OR peer OR peers OR community))	1837
9	#6 OR #7 OR #8	3458
10	#5 AND #9	281
11	Year in PROSPERO 2025,2024,2023	133

Trip Pro		
Search	Query	Results
1	(trial OR trials OR "clinical research") AND ("research coordinator" OR "research co ordinator" OR "trial coordinator" OR "trial co ordinator" OR recruiter* OR "subject recruitment" OR "participant recruitment" OR "patient recruitment" OR "research nurse" OR "trial	111

	nurse" OR accrual* OR underrepresent*) AND (navigator* OR "case manager" OR "case management" OR "care manager" OR "care management" OR promotora* OR "community matron" OR "guided care" OR "lay health worker" OR "community health worker" OR "village health worker" OR "outreach worker" OR facilitator* OR coach* OR advisor OR advisors OR "patient advocate" OR navigation* OR liaison* OR broker* OR educator* OR counselor* OR counsellor*), 2023-2025	

ICTRP (WHO)		
Search	Query	Results
1	("research coordinator" OR "research co ordinator" OR "trial coordinator" OR "trial co ordinator" OR recruiter OR recruitment OR enrollment OR enrolment OR "research nurse" OR "trial nurse" OR accrual OR underrepresented OR underrepresentation) AND (navigator OR "case manager" OR "case management" OR "care manager" OR "care management" OR promotora OR "community matron" OR "guided care" OR "lay health worker" OR "community health worker" OR "village health worker" OR "outreach worker" OR facilitator OR coach OR advisor OR advisors OR "patient advocate" OR navigation OR liaison OR broker OR educator OR counselor OR counsellor)	160

AHRQ via Google		
Search	Query	Results
1	("clinical research" OR trial) AND (coordinator OR recruiter OR recruitment OR underrepresented) AND (navigator OR "case manager" OR "case management" OR "care management" OR "guided care" OR "lay health worker" OR "community health worker" OR coach OR advisor OR advisors OR "patient advocate" OR navigation OR liaison OR broker OR counselor) site:effectivehealthcare.ahrq.gov/products - limited to 2023-present	8

Google Scholar		
Search	Query	Results
1	("clinical research" OR trial) AND (coordinator OR recruiter OR recruitment OR underrepresented) AND (navigator OR "case manager" OR "case management" OR "care management" OR "guided care" OR "lay health worker" OR "community health worker" OR coach OR advisor OR advisors OR "patient advocate" OR navigation OR liaison OR broker OR counselor) - limited to 2023-present, first 10 pages	99
